# Anammox Planctomycetes have a peptidoglycan cell wall

**DOI:** 10.1038/ncomms7878

**Published:** 2015-05-12

**Authors:** Muriel C.F. van Teeseling, Rob J. Mesman, Erkin Kuru, Akbar Espaillat, Felipe Cava, Yves V. Brun, Michael S. VanNieuwenhze, Boran Kartal, Laura van Niftrik

**Affiliations:** 1 Department of Microbiology, Institute for Water and Wetland Research, Faculty of Science, Radboud University, Nijmegen 6525AJ, The Netherlands; 2 Interdisciplinary Biochemistry Program, Indiana University, Bloomington, Indiana 47405, USA; 3 Department of Molecular Biology and Laboratory for Molecular Infection Medicine Sweden, Umeå Centre for Microbial Research, Umeå University, Umeå SE-90187, Sweden; 4 Department of Biology, Indiana University, Bloomington, Indiana 47405, USA; 5 Department of Chemistry, Indiana University, Bloomington, Indiana 47405, USA; 6 Department of Biochemistry and Microbiology, Laboratory of Microbiology, Gent University, Gent 9000, Belgium

## Abstract

Planctomycetes are intriguing microorganisms that apparently lack peptidoglycan, a structure that controls the shape and integrity of almost all bacterial cells. Therefore, the planctomycetal cell envelope is considered exceptional and their cell plan uniquely compartmentalized. Anaerobic ammonium-oxidizing (anammox) Planctomycetes play a key role in the global nitrogen cycle by releasing fixed nitrogen back to the atmosphere as N_2_. Here using a complementary array of state-of-the-art techniques including continuous culturing, cryo-transmission electron microscopy, peptidoglycan-specific probes and muropeptide analysis, we show that the anammox bacterium *Kuenenia stuttgartiensis* contains peptidoglycan. On the basis of the thickness, composition and location of peptidoglycan in *K. stuttgartiensis*, we propose to redefine Planctomycetes as Gram-negative bacteria. Our results demonstrate that Planctomycetes are not an exception to the universal presence of peptidoglycan in bacteria.

## Abstract (web summary)

Planctomycetes are unusual bacteria with complex intracellular compartments and an apparent lack of peptidoglycan in their cell walls. Here, van Teeseling *et al*. show that the cell wall of an anammox planctomycete does contain peptidoglycan, and propose to redefine planctomycetes as Gram-negative bacteria.

## Graphical abstract

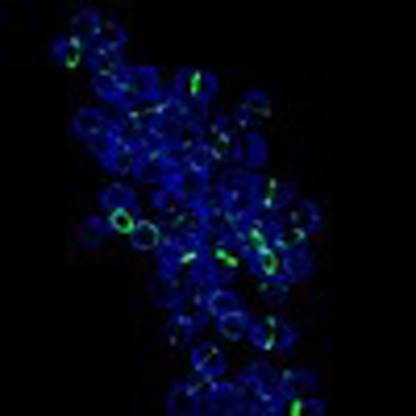

Maintaining cellular integrity is crucial for life and in particular challenging for small, unicellular organisms. In bacteria, the virtually universal solution to this problem is the presence of peptidoglycan, which also determines the cell shape and facilitates cell growth and division^1^. Peptidoglycan is a mesh-like heteropolymer consisting of a lysozyme-sensitive sugar backbone of alternating *N*-acetylglucosamine (GlcNAc) and *N*-acetylmuramic acid (MurNAc) residues cross-linked by short D-amino acid-rich peptide stems attached to each of the MurNAc residues^1^. Typically, the nascent peptide stem is a pentapeptide with the sequence of one L-alanine, one D-glutamate, one diamino acid and two D-alanines.

Traditionally, bacteria are classified into two groups based on their cell envelope properties. In Gram-positive bacteria, the peptidoglycan layer is relatively thick (15–30 nm), typically contains L-lysine (L-Lys) as the third amino acid and is located outside the cytoplasmic membrane, which is the outermost membrane of the cell^1^. The Gram-negative peptidoglycan is relatively thin (1.5–15 nm), typically contains *meso*-diaminopimelic acid (*meso*-DAP) instead of L-Lys and is located between the cytoplasmic and the Gram-negative specific outer membrane^1^.

Planctomycetes are extraordinary organisms that belong to the evolutionarily deep-branching bacterial superphylum of Planctomycetes, Verrucomicrobia and Chlamydiae^2^. Both Planctomycetes^3^,^4^,^5^ and Chlamydiae^6^ have been proposed to be among the few exceptions lacking peptidoglycan, which is one of the most conserved structural characteristics of bacteria. However, chlamydial species are commonly sensitive to antibiotics targeting peptidoglycan and have most of the genes involved in its biosynthesis, and the presence of peptidoglycan has recently been shown in some chlamydial species^7^,^8^. Paradoxically, although the free-living Planctomycetes are expected to need a peptidoglycan shell more than the intracellular and therefore osmotically protected Chlamydiae, they are usually insensitive to antibiotics targeting peptidoglycan^3^,^5^, are reported to lack a varying number of genes crucial for its biosynthesis^9^,^10^, and biochemical analyses failed to show the peptidoglycan components *meso*-DAP and MurNAc in isolated cell envelopes of all eight previously tested Planctomycete strains^3^.

Most planctomycete species, like typical Gram-negative bacteria, have two compartments enclosed by membranes. In Planctomycetes, the innermost membrane is unusually curved^11^. Historically, the planctomycetal outermost membrane was defined as a cytoplasmic membrane^11^,^12^. However, based on the bioinformatic analysis (focusing on marker genes for outer membrane protein and lipopolysaccharide insertion^13^), the outermost membrane has recently been proposed to be an outer membrane typical of Gram-negative bacteria^13^,^14^,^15^. On the basis of these hypotheses, Planctomycetes can be defined as either uniquely compartmentalized or Gram-negative bacteria. In the absence of any apparent peptidoglycan, arguments for the first hypothesis found considerable support^16^,^17^,^18^ and a shared evolutionary link between Planctomycetes and Eukaryotic cells was suggested^16^,^17^,^18^,^19^. Therefore, we argue that showing the presence of peptidoglycan in Planctomycetes and elucidating its characteristics would resolve the controversy concerning the Planctomycete-specific cell envelope and cell plan^11^,^12^,^13^,^14^,^15^,^20^ and its contentious link to the origins of eukaryotic cells^16^,^17^,^18^,^19^.

Anaerobic ammonium-oxidizing (anammox) bacteria form a distinct, phylogenetically deep-branching group within the Planctomycetes^9^. These microorganisms convert ammonium and nitrite to dinitrogen gas via nitric oxide and hydrazine as intermediates^21^. They occur in aquatic and terrestrial environments and play a crucial role in the biological nitrogen cycle estimated to produce approximately half of the dinitrogen gas present in the atmosphere^22^. Furthermore, the anammox process is widely applied to remove ammonium from wastewater^23^.

Compared with most other Planctomycetes, the anammox cell contains an additional, third, membrane-enclosed compartment^11^,^24^. This innermost compartment, the anammoxosome, harbours the catabolic machinery and is surrounded by the cytoplasm (also known as riboplasm), which contains the ribosomes and nucleoid^24^,^25^. Depending on the interpretation of the S-layer-enclosed outermost membrane^26^, the outermost compartment is either a unique cytoplasmic compartment called the paryphoplasm (in accordance with the historical definition) or a periplasm typical for a Gram-negative cell envelope. Unlike other Planctomycetes that divide by budding^9^,^16^, anammox bacteria divide by binary fission without the canonical cell division ring protein FtsZ^27^.

Similar to other Planctomycetes, the cell envelope of anammox bacteria was proposed to lack peptidoglycan. Since no anammox bacteria were included in the initial peptidoglycan-targeting biochemical analyses^3^, the absence of peptidoglycan in anammox bacteria was based on the absence of a peptidoglycan layer in the outermost anammox compartment of resin-embedded sections of either high-pressure frozen and freeze-substituted or chemically fixed anammox cells^11^,^28^. However, all genes essential for peptidoglycan biosynthesis are present in the genome of the anammox bacterium *Kuenenia stuttgartiensis*, apart from homologs of peptidoglycan-specific glycosyltransferase (GT) class 51 (CAZy database^29^) necessary to polymerize the sugar backbone. Despite the apparent lack of these GTs, it was recently shown^30^ that *K. stuttgartiensis* cells lyse with lysozyme in the presence of EDTA and their growth is inhibited by penicillin G.

Here we redefine the anammox Planctomycete *K. stuttgartiensis* as a Gram-negative bacterium based on the discovery and characterization of a peptidoglycan layer contained within the cell envelope. To this end, we use a complementary array of state-of-the-art techniques including continuous culturing, cryo-transmission electron microscopy (cryoTEM), incorporation of peptidoglycan-specific probes imaged with structured illumination microscopy (SIM) and ultrasensitive ultra performance liquid chromatography (UPLC)-based muropeptide analysis.

## Results

### CryoTEM shows hitherto unobserved cell envelope layer

**Figure 1:**
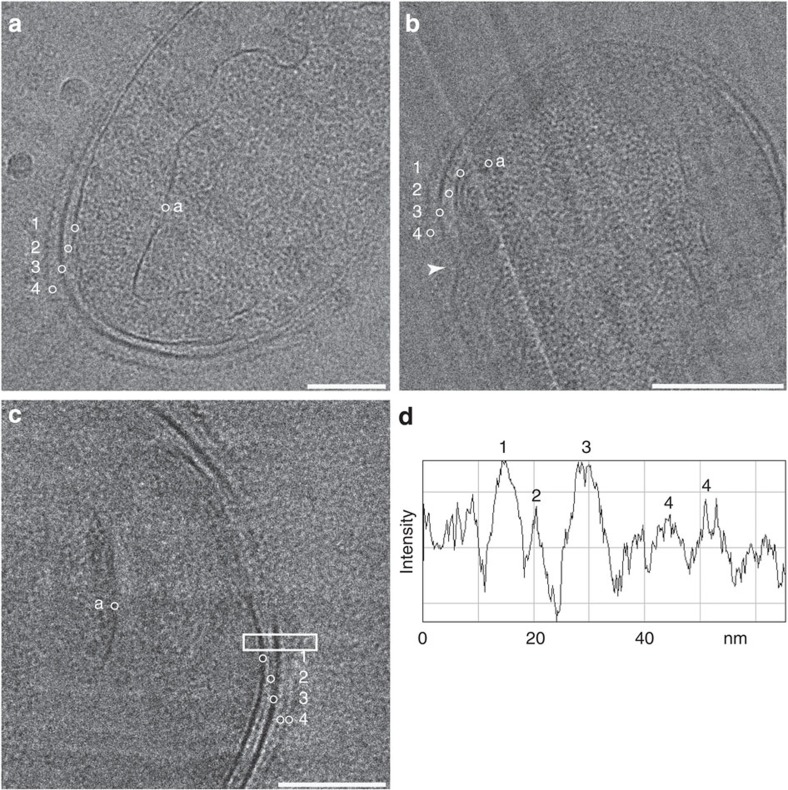
CryoTEM of vitreous sections revealed a previously unobserved layer in the cell envelope of *K. stuttgartiensis*. (**a**–**c**) Single *K. stuttgartiensis* cells as observed with cryo-electron microscopy of vitreous sections, in which all membranes and cell envelope layers have been annotated. (**b**) A dividing *K. stuttgartiensis* cell, the division site is indicated with an arrowhead. (**d**) The intensity profile of the area in the box in (**c**) encompassing the cell envelope verified the extra layer to be a separate entity. a, anammoxosome membrane; 1, cytoplasmic membrane; 2, putative peptidoglycan; 3, outer membrane; 4, S-layer. Scale bars, 100 nm.

To study the cell envelope of *K. stuttgartiensis*, we used a highly enriched (>95%) culture of free-living, planktonic cells. Cryo-electron microscopy of vitreous sections^31^, reflecting the near-native hydrated state of the cells, showed a layer in the cell envelope that was previously unobserved. This electron-dense layer was located underneath the outermost membrane (Fig. 1). The thickness of the layer varied between 4.5 and 6 nm, which is in the range reported for peptidoglycan in Gram-negative bacteria^1^. The location of this layer, in the outermost cell compartment, matched the location of peptidoglycan in Gram-negative bacteria and therefore this layer was a good candidate to represent the peptidoglycan shell in this bacterium.

### Peptidoglycan isolation yields lysozyme-sensitive sacculi

**Figure 2:**
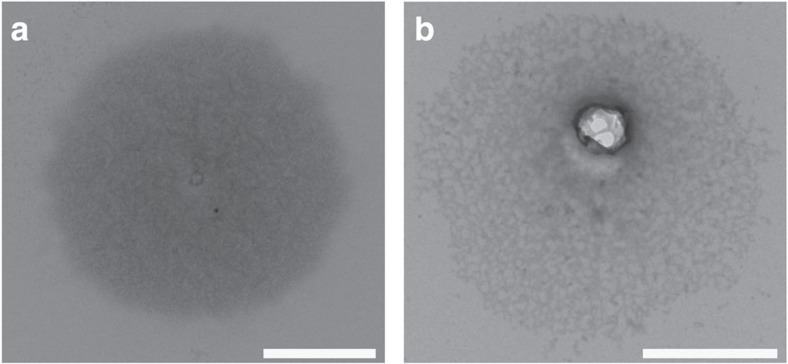
Lysozyme-sensitive sacculi were obtained by boiling *K. stuttgartiensis* cells enriched by density centrifugation in SDS. (**a**) TEM of *K. stuttgartiensis* sacculus using negative staining. (**b**) After lysozyme treatment, the *K. stuttgartiensis* sacculi were absent or had a fibrous appearance, as observed by negative staining via TEM. Scale bars, 1 μm.

After they were harvested from an enrichment culture (>95%), *K. stuttgartiensis* cells were further enriched to 99.9% purity using density centrifugation. When this highly enriched sample was boiled in SDS and negatively stained with uranyl acetate, cell-shaped structures similar to peptidoglycan sacculi were recovered (Fig. 2a, Supplementary Fig. 1). In rare instances, sacculi with a figure-eight shape, probably obtained from dividing *K. stuttgartiensis* cells, were also detected (Supplementary Fig. 2). When *K. stuttgartiensis* sacculi were incubated for 5 h with lysozyme, which cleaves peptidoglycan by hydrolysing the β-1,4 bonds between the two sugar components MurNAc and GlcNAc^32^, the sacculi disintegrated and only fibrous material was visible (Fig. 2b). This was consistent with the previous observation of fibrous material upon enzymatically cleaving the sugar backbone of *Escherichia coli* peptidoglycan sacculi^33^. The disintegration of the sacculi was solely dependent on the addition of lysozyme, as sacculi incubated without lysozyme remained intact. This suggested that the sacculi were indeed composed of a peptidoglycan-like sugar backbone. Interestingly, this observation also indicated that *K. stuttgartiensis* must be encoding and expressing at least one polymerizing GT class 51 enzyme or another unknown enzyme that polymerizes the peptidoglycan sugar backbone. The apparent absence of such a gene from the *K. stuttgartiensis* genome could be due to the fact that the genome is currently only ⩾98% complete^34^. However, according to the CAZy database^29^ other Planctomycetes, as well as Chlamydiae^6^, also lack GT class 51 enzymes and therefore it seems more likely that the enzyme is a member of a new, perhaps Planctomycetes- and Chlamydia-specific, uncharacterized class of GTs.

### Peptidoglycan peptide stem was verified by specific probes

**Figure 3:**
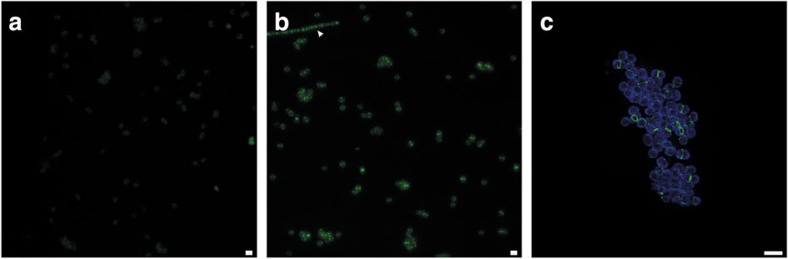
Fluorescence microscopy of *K. stuttgartiensis* cells grown in the presence of peptidoglycan-specific D-alanine dipeptide probes indicated the presence of peptidoglycan. (**a**) The negative control probe ELA-LA, which cannot be incorporated, shows only a faint background. (**b**) Septal incorporation of EDA-DA is present both in anammox and rod-shaped non-anammox species present in the bioreactor (arrowhead). (**c**) SIM clearly shows that EDA-DA was incorporated specifically at the cell division site. Probe incorporation was visualized with a complimentary fluorophore using click chemistry (green) and cell surfaces were labelled by amine-reactive Pacific Blue *N*-hydroxysuccinimide ester (NHS) (blue). ELA-LA, ethynyl-L-alanyl-L-alanine. Scale bars, 2 μm.

The peptidoglycan biosynthesis machinery is promiscuous^35^ and therefore non-canonical D-amino acid compounds, such as fluorescent or bio-orthogonal D-amino acids or bio-orthogonal D-amino acid containing dipeptides, can be incorporated at the C-terminus of the peptide stems. This has recently been exploited to specifically stain peptidoglycan^8^,^36^,^37^. When grown for a part of the cell division cycle (10–14%) in the presence of the bio-orthogonal D-amino acid dipeptide ethynyl-D-alanyl-D-alanine (EDA-DA), *K. stuttgartiensis* cells displayed the characteristic fluorescent signal of probe incorporation after coupling with a fluorophore (Fig. 3b,c). Comparable to peptidoglycan labelling observed in other microorganisms^36^, most of the D-amino acid dipeptide incorporation occurred at the cell division site as determined by fluorescence and super-resolution SIM, while experiments with L-amino-acid dipeptide controls only resulted in background fluorescence (Fig. 3a).

### Muropeptide analysis confirms sacculi contain peptidoglycan

**Figure 4:**
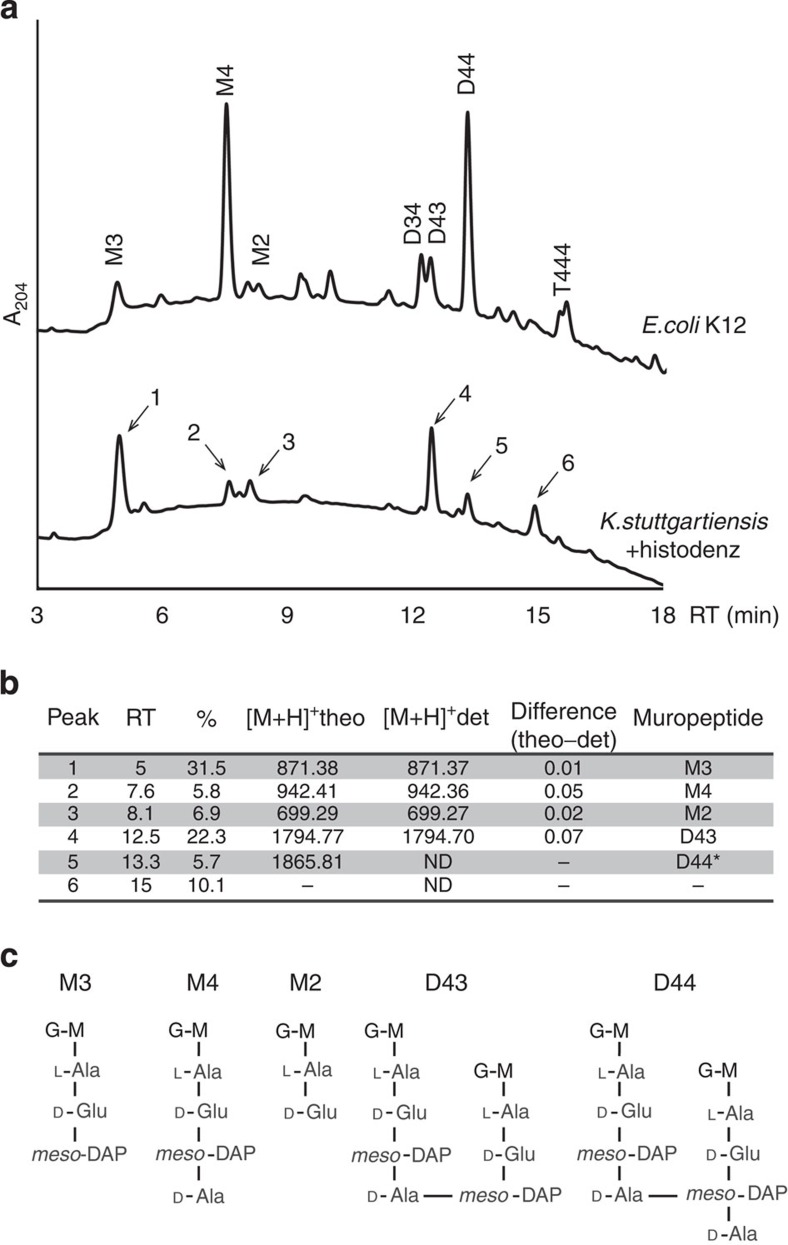
Structural characterization by UPLC and mass spectrometry of the peptidoglycan present in *K. stuttgartiensis*. (**a**) UPLC analyses of *K. stuttgartiensis* peptidoglycan. *E. coli* K12 PG profile is included as a reference. Peaks labelled 1–6 correspond to muropeptides of *K. stuttgartiensis* Histodenz-enriched cultures. RT, retention time; A_204_, absorbance 204 nm. (**b**) Mass analysis of the PG peptidoglycan subunits isolated in panel A by MALDI-TOF. *, D44 was formulated based on its similar retention time with *E. coli* D44 R.T; ND, not determined; %, relative abundance; theo, theoretical mass; det, determined mass. (**c**) Schematic representation of *K. stuttgartiensis* detected PG species in their reduced state. M2, *N*-acetylglucosamine-(GlcNAc)-*N*-acetylmuramic acid (MurNAc)-L-Ala-D-Glu; M3, GlcNAc-MurNac-L-Ala-D-Glu-*meso*-DAP; M4, GlcNac-MurNAc-L-Ala-D-Glu-*meso*-DAP-D-Ala; D43, dimer muropeptide of a M4 D,D-cross-linked to a M3; M, MurNAc; G, GlcNAc.

The structure of the peptidoglycan subunits (muropeptides) was determined by UPLC and subsequent mass spectrometry (Fig. 4a). Muropeptides were obtained by muramidase degradation of sacculi prepared from Histodenz-purified *K. stuttgartiensis* cells (99.9%). Indeed, GlcNAc-MurNAc coupled to 2–4 amino acids (M2–M4) as well as cross-linked fragments (D43 and presumably D44) were detected in *K. stuttgartiensis* sacculi (Fig. 4b,c). The largest monomeric peptidoglycan fragment obtained (M4) was identified as *GlcNac-MurNAc-L-Ala-D-Glu-meso-DAP-D-Ala*. Strikingly, the third amino acid was identified as *meso*-DAP (Fig. 4), which is typical for Gram-negative peptidoglycan^1^. In agreement with this observation, all genes necessary for *meso*-DAP synthesis are present in the *K. stuttgartiensis* genome (Supplementary Table 1). Furthermore, the predicted protein sequence for MurE, which couples the third amino acid to the peptidoglycan precursors, has the arginine residue (Arg416 in *E. coli*) necessary for specifically incorporating *meso*-DAP (instead of L-Lys) into the peptidoglycan precursor^38^.

Peptidoglycan from *K. stuttgartiensis* had relatively little M4 and D44 compared with *E. coli* K12. In *K. stuttgartiensis*, M3 was the most abundant monomer, which suggested that the enzyme cleaving between the third (*meso*-DAP) and fourth (D-Ala) amino acid in the peptide stem (L,D-endopeptidase) was highly active. Further investigation will be necessary to determine whether the abundance of M3 over M4 has an impact on the biology of the bacterium. The presence of D43 cross-linked muropeptides indicated the expression of D,D-transpeptidases, which was supported by the identification of homologs to Pbp2 and Pbp3 encoded in the genome of *K. stuttgartiensis* (Supplementary Table 1).

## Discussion

This study presents the first experimental evidence of peptidoglycan in an anammox Planctomycete. We show that *K. stuttgartiensis* has a relatively thin, *meso*-DAP-containing peptidoglycan layer located underneath its outer membrane, which fits to the typical characteristics of Gram-negative peptidoglycan. The location of the peptidoglycan in anammox bacteria is striking since the non-FtsZ containing cell division ring^27^ was observed in the exact same compartment of the cell. To the best of our knowledge, a cell division ring has not been previously observed in the peptidoglycan-containing compartment of any bacterium and this further substantiates that anammox bacteria divide via a unique mechanism. On the basis of our investigations, facilitated by new methodology that recently became available, and the results of Jeske *et al*.^39^ that present similar findings in other planctomycetal lineages, we propose to redefine the Planctomycetes as Gram-negative microorganisms and therefore end the longstanding controversy about the planctomycetal cell plan. Taken together, these findings clearly show that Planctomycetes are not an exception to the universal rule of peptidoglycan cell walls in bacteria, and consequently all free-living bacteria possess peptidoglycan. These findings also lead us to conclude that an evolutionary link between Planctomycetes and eukaryotes is unlikely.

## Methods

### *K. stuttgartiensis* enrichment culture

Free-living planktonic *K. stuttgartiensis* cells were grown in an enrichment culture (∼95% *K. stuttgartiensis*) in a membrane bioreactor as described previously^40^. In short, the reactor (working volume 11 l) was fed continuously with mineral medium^41^ containing 45 mM nitrite and ammonium (each) at a flow rate of 4.2 ml min^−1^ (∼6 l per day). The pH of the reactor was controlled at 7.3 with a potassium bicarbonate solution (100 g l^−1^). The reactor was operated at 33 °C and was stirred at 300 r.p.m. A gas mixture of Ar/CO_2_ (95/5%) with a flow of 10 ml min^−1^ was supplied to the reactor to maintain anaerobic conditions. The cells were washed out the reactor continuously (1.1 l per day) to maintain the cell density of the culture (optical density at 600 nm was 1.1–1.2).

### Cryo-transmission electron microscopy

*K. stuttgartiensis* cells (4 ml) were pelleted (4 min, 600*g*, 33 °C) and resuspended in a small amount (50 μl) of mineral medium containing 1.25 g l^−1^ sodium bicarbonate and trace minerals^41^ and left to recover for 15 min at 33 °C. Cells were gently mixed with an equal volume of 40% dextran (70 kD molecular weight) in mineral medium^41^, loaded in copper tubes and high-pressure frozen (HPM100 (Leica Microsystems, Vienna, Austria)). For preparing vitreous sections, copper tubes were loaded in a cryo-ultramicrotome (Leica FC7/UC7, Leica Microsystems) pre-cooled at −145 to −150 °C. Tubes were trimmed to a pyramid with a 50 by 50 μm block face using a cryo-trim 20° diamond knife (Diatome, Biel, Switzerland). Frozen-hydrated sections (45 nm) for cryo-electron microscopy of vitreous sections were sectioned on a 30° cryo-immuno diamond knife (Diatome). Sections were attached to carbon-coated 200 mesh copper grids (Stork-Veco, Eerbeek, Netherlands) by electrostatic force using the Crion system (Leica Microsystems). Grids were transferred to the loading station of a high-tilt cryotomography holder (914 (Gatan, Munich, Germany)) under liquid nitrogen. Sections were imaged in a JEOL (Tokyo, Japan) JEM 2100 Transmission Electron Microscope operated at 200 kV in low-dose mode. Images were recorded using the Gatan F4000 bottom-mount camera.

### Isolation of peptidoglycan sacculi

*K. stuttgartiensis* cells were further enriched from the other microorganisms in the enrichment culture by density centrifugation (15 min, 1,000*g*) on a 15–30% Histodenz gradient in 20 mM HEPES buffer pH 7.5 including 1 g l^−1^ sodium bicarbonate. A distinct red band indicating the presence of *K. stuttgartiensis* was present approximately halfway through the gradient. Here *K. stuttgartiensis* presence and abundance was verified by fluorescence *in situ* hybridization microscopy using the specific Amx820 (ref. 42) and Pla46 (ref. 43) probes. Cells were boiled in 4% SDS for 60 min at 100 °C and applied to a glow-discharged formvar-carbon-coated copper grid. After 20 min, negative staining was performed by washing the grids once in 0.5% uranyl acetate in MQ, staining (60 s) on 0.5% uranyl acetate and washing on three drops of MilliQ. Sacculi were visualized in a JEOL 1010 TEM operating at 60 kV.

To investigate whether the observed sacculi consisted of peptidoglycan, sacculi (obtained from cells taken directly from the enrichment reactor) were incubated (5 h, 37** **°C) with lysozyme (from chicken egg white; 10 mg ml^−1^; with and without 20 mM EDTA) before visualizing via negative staining as described above. As a negative control, sacculi were incubated without lysozyme.

### Incorporation of peptidoglycan-specific probe EDA-DA

Cells (100 ml) harvested from the membrane bioreactor were concentrated and resuspended in 5 ml 20 mM HEPES buffer pH 7.8 including 1.25 g l^−1^ sodium bicarbonate. Afterwards, cell suspensions were made anoxic by applying under-pressure and flushing with Ar/CO_2_ (95%/5%) ten times. Then the cell suspensions were transferred to a 100 ml fed-batch reactor inside an anaerobic chamber with an Ar/H_2_ (95%/5%) atmosphere. O_2_ in the Ar in the anaerobic chamber was removed by passing Ar over a Pd catalyst (0.2 p.p.m. residual O_2_). The cells were supplied with the above-mentioned HEPES buffer containing 7 mM NH_4_^+^ and NO_2_^−^ each and the peptidoglycan-specific probe EDA-DA (1 mM) with a flow rate of 5 ml h^−1^ for 12–15 h. The fed-batch reactors were stirred at 150 r.p.m. and incubated in the dark at 30 °C. As a control, cells were grown under the same conditions in the presence of ethynyl-L-alanine-L-alanine (ELA-LA) (1 mM). Cells were harvested, washed three times in the above-mentioned HEPES buffer (3,000*g*, 10 min) and resuspended in 4 ml HEPES buffer. The resuspended cells were put in an equal volume of 4% paraformaldehyde in the HEPES buffer and incubated for 20 min at room temperature and subsequently for 90 min at 4 °C. The fixed cells were pelleted by centrifugation (11,700*g*, 5 min) and resuspended in 0.5 ml 0.1% paraformaldehyde for temporary storage. Pelleted cells grown in the presence of EDA-DA and ELA-LA. were permeabilized by incubating them for 5 min in 1.5 ml PBS with 0.25% Triton X-100. The cells were washed once in PBS, and the fluorophore Alexa 488-azide (20 μM) was attached via a copper(I) catalysed click reaction (incubation 60 min in 1.5 ml at room temperature in the Click-iT Cell Reaction Buffer Kit (ThermoFischer, Waltham, USA). Afterwards, cells were washed three times in PBS (1.5 ml), incubated 20 min at room temperature in the presence of 5 μg ml^−1^ Pacific Blue NHS ester (ThermoFischer, Waltham, USA), washed twice in PBS and visualized via fluorescence microscopy or SIM as described previously^36^. In short, images were collected with a DeltaVision (GE Healthcare, Pittsburgh, USA) OMG Imaging System equipped with a Photometrics (Tucson, USA) Cascade II EMCCD camera (excitation: 405 nm and emission: 419–465 nm).

### Analysis of muropeptides obtained from sacculi

Sacculi were prepared from the *K. stuttgartiensis* cells (0.8 l, OD_600_ 1.1) enriched by density centrifugation as described above. Peptidoglycan was isolated largely as described before^35^. Briefly, cell pellets were boiled in 4% SDS for 60 min at 100 °C. After boiling for an additional 4 h, sacculi were further stirred overnight at 37 °C and then, SDS was washed out by ultracentrifugation. Sacculi were resuspended in 200 μl of 50 mM sodium phosphate buffer pH 4.5 and digested overnight with 30 μg ml^−1^ muramidase (cellosyl, Hoechst) at 37 °C. Muramidase digestion was stopped by incubation in a boiling water bath (5 min). Coagulated protein was removed by centrifugation. The supernatants were mixed with 150 μl 0.5 M sodium borate pH 9.5, and subjected to reduction of muramic acid residues into muramitol by sodium borohydride treatment (10 mg ml^−1^ final concentration, 30 min at room temperature). Samples were adjusted to pH 3.5 with phosphoric acid. UPLC analyses of muropeptides were performed on an ACQUITY UPLC BEH C18 Column, 130 A, 1.7, 2.1 and 150 mm (Waters, USA) and detected at 204 nm. Muropeptides were separated using a linear gradient from buffer A (phosphate buffer 50 mM, pH 4.35) to buffer B (phosphate buffer 50 mM, pH 4.95 methanol 15% (v/v)) in 20 min. Muropeptide purification was performed by HPLC on an Aeris peptide column (250 × 4.6 mm; 3.6 μm particle size; Phenomenex, USA). The identity of individual muropeptides was established by MALDI-TOF (Voyager DE-STR).

## Author contributions

M.C.F.v.T., R.J.M., B.K. and L.v.N. designed the project; R.J.M. performed the cryoTEM experiments. M.C.F.v.T. isolated sacculi and performed the lysozyme experiment; M.C.F.v.T. and B.K. set-up fed-batch incubations with D-amino acid probes; E.K., Y.V.B. and M.S.V. provided the D-amino acid probes; E.K. performed click chemistry and visualization of the incorporation experiments (fluorescence microscopy and SIM); M.C.F.v.T., R.J.M. and A.E. prepared sacculi for muropeptide analysis; A.E. performed UPLC and MS analysis; A.E. and F.C. analysed the muropeptides; M.C.F.v.T., R.J.M., E.K., A.E., F.C., Y.V.B., M.S.V., B.K. and L.v.N. analysed data; M.C.F.v.T., R.J.M., B.K. and L.v.N. wrote the paper with input from all authors.

## Additional information

**How to cite this article:** van Teeseling, M. C. F. *et al*. Anammox Planctomycetes have a peptidoglycan cell wall. *Nat. Commun.* 6:6878 doi: 10.1038/ncomms7878 (2015).

## Supplementary Material

Supplementary Information Supplementary Figures 1-2 and Supplementary Table 1
