# Supplementary material for: Anammox Planctomycetes have a peptidoglycan cell wall
Source: Nat Commun. 2015 May 12;6:6878. doi: 10.1038/ncomms7878 (PMC4432595; doi:10.1038/ncomms7878)
Supplement: Supplementary Information — Supplementary Figures 1-2 and Supplementary Table 1 [file ncomms7878-s1.pdf]

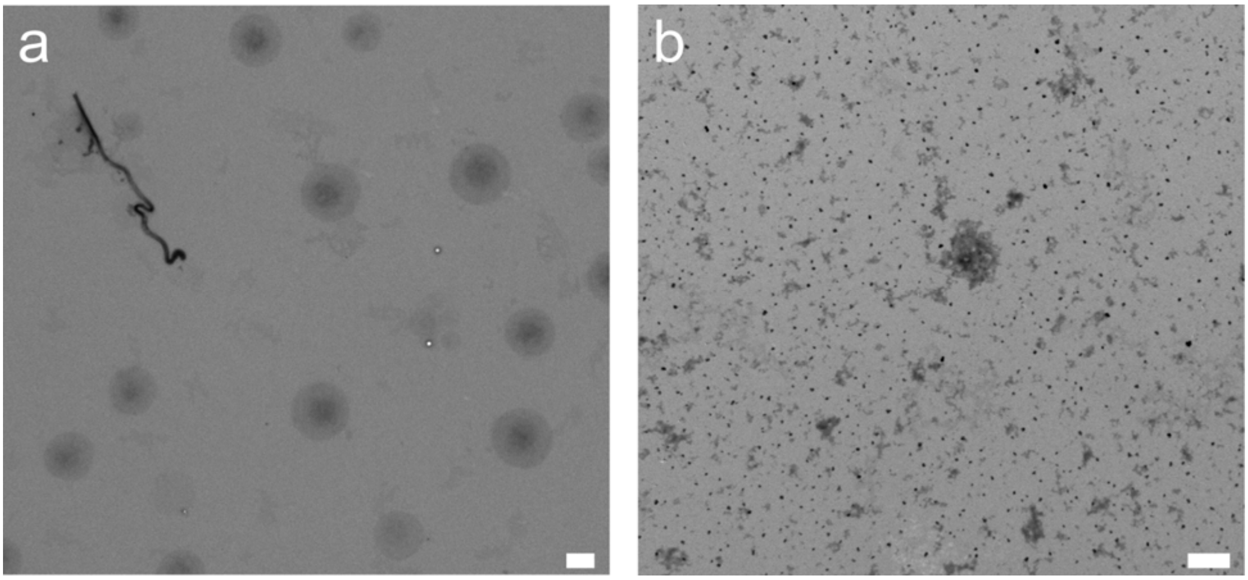

Supplementary Figure 1: Lysozyme-sensitive sacculi were obtained by boiling *K. stuttgartiensis* cells, without enrichment by density centrifugation, in SDS. (a) An overview of a grid after negative staining shows multiple thin sacculi of *K. stuttgartiensis* (round) and a thicker sacculus from a long rod shaped cell present in the reactor alongside *K. stuttgartiensis*. (b) After lysozyme treatment the *K. stuttgartiensis* sacculi were absent or had a fibrous appearance, as observed by negative staining via TEM. Scale bars 1 µm.

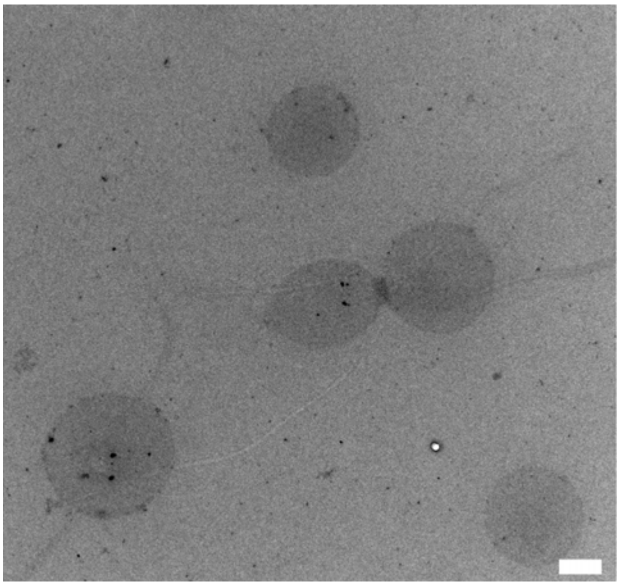

Supplementary Figure 2: Some lysozyme-sensitive sacculi obtained by boiling *K. stuttgartiensis* cells, without enrichment by density centrifugation, in SDS appear to stem from dividing cells. A figure-eight shaped sacculus, probably obtained from a dividing *K. stuttgartiensis* cell surrounded by sacculi from non-dividing *K. stuttgartiensis* cells, as observed by negative staining via TEM. Scale bar 1 µm.

Supplementary Table 1: Predicted peptidoglycan biosynthesis associated proteins in *K. stuttgartiensis*. In silico identification of *K. stuttgartiensis* peptidoglycan associated proteins based on NCBI protein BLAST tool analysis using *E. coli* homologs as source, using a cutoff of 1.10^{-5}. Proteins highlighted in white are homologous to *E. coli* cytoplasmic synthesis proteins. Proteins in grey and light grey are homologous to *E. coli* proteins with periplasmic activities. Highlighted in grey are proteins with D,D- and L,D-transpeptidase activity and in light grey proteins with peptidoglycan hydrolysis activities. Proteins in dark grey correspond to homologous proteins involved in the biosynthetic pathways of meso-DAP.

E. coli peptidoglycan related proteins	*K. stuttgartiensis* locus (NCBI ID)	Kust number	Query cover	E value	Identity
MurA	emb|CAJ74073.1|	kuste3313	98%	3.00E-122	48%
MurB	emb|CAJ74091.1|	kuste3330	96%	9.00E-16	25%
MurC	emb|CAJ73131.1| emb|CAJ71429.1|	kuste2385<br>kustc0684	93%<br>90%	2E-86<br>1E-57	36%<br>31%
MurD	emb|CAJ74243.1|	kuste3480	87%	3.00E-45	30%
MurE	emb|CAJ73124.1|	kuste2378	94%	4.00E-88	36%
MurF	emb|CAJ73125.1|	kuste2379	99%	1.00E-69	31%
Ddl	emb|CAJ73132.1|	kuste2386	99%	4.00E-79	41%
Alr	emb|CAJ73156.1|	kuste2410	98%	8.00E-56	34%
MurI	emb|CAJ72251.1|	kustd1506	88%	2.00E-24	30%
MraY	emb|CAJ73126.1|	kuste2380	93%	5.00E-104	44%
MurG	emb|CAJ73129.1|	kuste2383	97%	7.00E-43	28%
MreB	emb|CAJ72643.1|	kustd1898	98%	4.00E-119	54%
Pbp2	emb|CAJ72640.1|	kustd1895	91%	8.00E-58	26%
Pbp3	emb|CAJ73122.1|	kuste2376	91%	3.00E-88	32%
Ynhg	emb|CAJ75008.1|	kuste4246	56%	4.00E-14	29%
Ycfs	emb|CAJ75008.1|	kuste4246	58%	1.00E-06	27%
Pbp4	emb|CAJ74399.1|	kuste3636	92%	6.00E-40	25%
MltA	emb|CAJ70755.1|	kusta0010	68%	2.00E-32	33%
Dap epi	emb|CAJ72320.1|	kustd1575	98%	2.00E-55	38%
DapA	emb|CAJ72115.1|	kustd1370	100%	7.00E-93	49%
DapB	emb|CAJ71744.1|	kustc0999	96%	1.00E-64	42%
DapE	emb|CAJ73921.1|	kuste3163	82%	1.00E-16	25%
DapF	emb|CAJ72320.1|	kustd1575	98%	2.00E-54	38%
